# Editorial: Mental health needs and access to care among LGBTQ+ individuals

**DOI:** 10.3389/fpubh.2026.2016876

**Published:** 2026-10-21

**Authors:** Christopher Lance Coleman, Ronald Piscotty, Joachim Voss

**Affiliations:** 1 Howard University College of Nursing and Allied Health Sciences, Washington, DC, United States; 2 Oakland University School of Nursing, Rochester, MI, United States; 3 University of Nebraska Medical Center College of Nursing, Omaha, NE, United States

**Keywords:** adults, equity, global mental health, LGBTQ+, mental health

LGBTQ+ individuals experience disproportionate mental health challenges shaped by stigma, discrimination, and persistent gaps in access to culturally competent care. This underscores the need for sustained scientific inquiry that not only documents disparities but also identifies mechanisms of risk, strengthens protective factors, and informs interventions that promote psychological wellbeing. The purpose of this Research Topic is to contribute rigorous, interdisciplinary scholarship that advances our understanding of LGBTQ+ mental health across diverse global contexts and supports the development of more equitable systems of care. The nine articles included in this Research Topic reflect the breadth and depth of contemporary LGBTQ+ mental health scholarship. They span multiple countries, methodological approaches, and interdisciplinary perspectives, illustrating the global nature of both the challenges and opportunities for mental health interventions. Together, they offer a multifaceted perspective of the sociocultural, structural, and psychological factors that shape mental health outcomes for LGBTQ+ populations.

Several articles highlight the central role of minority stress and intersectional experiences in shaping mental health outcomes. Liu's thematic analysis of lesbian and bisexual cisgender women in Chinese higher education provides a nuanced examination of how stigma, invisibility, and cultural expectations intersect to produce psychological distress. Zhang et al.'s study of identity centrality among Chinese sexual minority students further demonstrates how identity-related stressors can function as both protective and risk-enhancing mechanisms depending on context. These studies collectively emphasize the importance of including the sociocultural context when examining LGBTQ+ mental health. Endres and colleagues extend Liu's work by documenting the relationship between psychosocial distress and discrimination among university students. Their findings underscore the need for institutionalized supportive climates that actively counteract stigma and promote inclusion. Educational settings are critical developmental environments, and when they fail to affirm LGBTQ+ identities, they can become sources of chronic stress that reverberate across academic, social, and psychological domains.

Structural and systemic factors emerge as equally important determinants of mental health. Meneguzzo et al.'s analysis of psychiatric care for transgender individuals in Italian prisons reveals profound institutional gaps that impede access to affirming treatment. Their work highlights how prison systems lack the policies, training, and resources necessary to support transgender individuals, resulting in unmet mental health needs and heightened vulnerability for this population. Kreysa's conceptual framework expands this structural perspective by illustrating how LGBTQ+ individuals navigate decision-making across health, housing, financial, and service domains, areas where inequities often accumulate and affect wellbeing. This framework underscores the interconnectedness of social systems and the importance of addressing structural barriers holistically rather than in isolation. Theoretical advancement is reflected in Angelene and Alamelu's extended theory of trans-identity, which reframes gender euphoria as a central construct. By shifting focus from distress to positive identity experiences, this theory offers new directions for psychological research and clinical practice. It encourages scholars and practitioners to consider how joy, affirmation, and embodiment contribute to mental health and resilience among transgender individuals. Several contributions identify promising avenues for expanding research among sexual minority students. Oliveira da Cruz et al. explore sports participation as a potential buffer against anxiety and depression among sexual minority students. Their data suggest that inclusive community environments, particularly those that promote physical activity and social connection, may serve as meaningful protective factors against mental distress. Structural and systemic factors also emerge as critical determinants of mental health. Meneguzzo et al.'s analysis of psychiatric care for transgender individuals in Italian prisons highlights the importance of considering physiological and behavioral factors alongside social and identity-related stressors.

Complementing these empirical insights, Mathews et al. emphasize the need to prepare nursing students to deliver culturally competent mental healthcare. Their narrative review underscores that expanding access to affirming services requires not only policy change but also workforce development. Training future clinicians to understand LGBTQ+ identities, experiences, and needs is essential to reduce disparities and improve care quality.

In summary, this Research Topic contributes important data that advances the field of mental health among LGBTQ+ individuals by illuminating barriers, broadening theoretical and empirical understanding, and highlighting innovative strategies that support LGBTQ+ mental health. The contributing authors reflect the commitment of mental health scholars to generate evidence that informs practice, policy, and strengthens psychological wellbeing. As research continues to evolve, the contributions in this Research Topic underscore the importance of sustained, interdisciplinary engagement in addressing mental health disparities among LGBTQ+ individuals. The global scope of the included articles demonstrates that while cultural contexts differ, many challenges faced by LGBTQ+ individuals are shared globally.

We hope this Research Topic stimulates further inquiry, fosters collaboration across fields, and supports the development of environments where LGBTQ+ individuals can thrive. The evidence presented here affirms that advancing LGBTQ+ mental health is not only a scientific imperative but also a matter of advancing equity, dignity, and human rights. Continued research, advocacy, and innovation are essential for building systems of care that recognize the full diversity of LGBTQ+ lives and experiences.

